# *Aeromonas dhakensis*: A Zoonotic Bacterium of Increasing Importance in Aquaculture

**DOI:** 10.3390/pathogens13060465

**Published:** 2024-05-31

**Authors:** Kerry L. Bartie, Andrew P. Desbois

**Affiliations:** 1 Faculty of Health Sciences and Sport, University of Stirling, Stirling FK9 4LA, UK; k.l.bartie1@stir.ac.uk; 2 Institute of Aquaculture, University of Stirling, Stirling FK9 4LA, UK

**Keywords:** aeromonad virulence, antimicrobial resistance, bacteria identification, molecular epidemiology, motile *Aeromonas* septicaemia (MAS), zoonosis

## Abstract

*Aeromonas dhakensis* is increasingly recognised to be an important pathogen responsible for disease losses in warm-water aquaculture and, similar to several other *Aeromonas* species, it can infect humans. Knowledge of *A. dhakensis* is accumulating, but this species remains relatively under-investigated compared to its close relative, *Aeromonas hydrophila.* The significance of *A. dhakensis* may have been overlooked in disease events of aquatic animals due to issues with reliable identification. Critical to appreciating the importance of this pathogen is the application of dependable molecular tools that enable accurate identification and discrimination from *A*. *hydrophila* and other motile aeromonads. This review aims to synthesise the key literature on *A. dhakensis*, particularly with relevance to aquaculture, including knowledge of the bacterium derived from disease case studies in aquatic hosts. Identification methods and strain phylogeny are discussed, with accurate detection important for prompt diagnosis and for distinguishing strains with heightened virulence. Increasing evidence suggests that *A. dhakensis* may be more virulent than *A. hydrophila* and correct identification is required to determine the zoonotic risks posed, which includes concerns for antibiotic-resistant strains. This review provides an impetus to improve species identification in the future and screen strain collections of presumptive *Aeromonas* spp. retrospectively to reveal the true prevalence and impact of *A. dhakensis* in aquaculture, the environment, and healthcare settings.

## 1. Introduction

Aeromonad bacteria are ubiquitous inhabitants of the aquatic environment, with several species considered opportunistic pathogens of humans and animals [1,2]. In humans, motile *Aeromonas* spp. cause gastroenteritis, infections of wounds, skin and soft tissues and bacteraemia, with the most commonly isolated species being *Aeromonas caviae*, *Aeromonas dhakensis*, *Aeromonas hydrophila* and *Aeromonas veronii* biovar *sobria* [2,3,4]. Meanwhile, several species, notably *A. hydrophila*, *A. dhakensis*, *A. veronii*, *Aeromonas jandaei*, *Aeromonas sobria* and *A. caviae*, are responsible for motile *Aeromonas* septicaemia (MAS) syndrome in many species of fish, a debilitating condition implicated in significant morbidity and mortality losses in warm-water intensive aquaculture [5,6,7,8,9,10,11,12]. MAS has a varying presentation across hosts, with typical external signs in fish including reddened or eroded fins, diffuse haemorrhages on the skin, eyes and fins, scale loss and appearance of surface lesions, distension of the abdomen, inflammation of the anus and exophthalmia [13,14,15]. Internal signs include blood in the body cavity, haemorrhages and reddening of muscle tissues and intestines and swelling of the spleen and kidney [13,14,15]. When a MAS outbreak occurs, chronic ulcerative lesions observed in the population can progress rapidly to haemorrhagic septicaemia and mass mortality. Of the pathogenic aeromonad species, *A. dhakensis* has been relatively neglected in terms of study, but this species has gained increased attention recently for its role in human clinical cases [1,2] and MAS outbreaks [16,17]. Indeed, *A. dhakensis* has been isolated from aquatic hosts worldwide, such as in Asia, Europe and Central and South America, including from internal organs of hosts showing signs of disease (Table 1). In addition to infecting fish and humans, *A. dhakensis* isolates have been cultivated from marine mammals [18,19], rodents [20,21], reptiles [22,23] and invertebrates [24,25,26]. Moreover, microbiological screens have detected *A. dhakensis* in otherwise healthy populations of fish [7] and people [27], various aquatic environments including river water [28,29], lagoons [30], drinking water [31] and wells [32], as well as sewage [33] and foodstuffs [34,35,36].

Similar to the recent reviews of *A. dhakensis* in human disease [1,54], this present review aims to address gaps in the knowledge and understanding of this pathogen and raise awareness of its significance in aquatic animals, such that problems can be appreciated and suitably addressed. A key focus is methods for reliable identification because misidentification, often as *A. hydrophila*, may explain, at least in part, the lack of recognition for *A. dhakensis* in aquatic disease events. This article also considers virulence, current treatment and prevention strategies, potential risks to human health and concerns posed by antibiotic-resistant strains. The review provides an impetus to improve species identification in the future and to screen existing strain collections to reveal the true prevalence and impact of *A. dhakensis* in aquatic systems.

## 2. Taxonomy and Identification

### 2.1. Nomenclature and Classification

**Figure 1:**
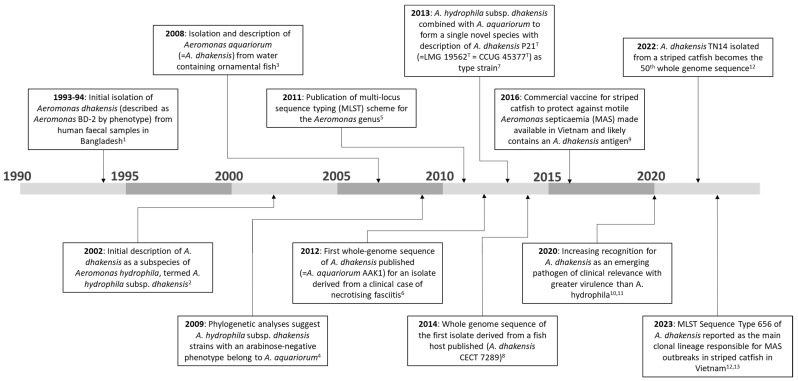
A timeline of selected milestones for *Aeromonas dhakensis* research. ^1^ Kühn et al. [55]; ^2^ Huys et al. [56]; ^3^ Martınez-Murcia et al. [48]; ^4^ Martínez-Murcia et al. [57]; ^5^ Martino et al. [62]; ^6^ Wu et al. [63]; ^7^ Beaz-Hidalgo et al. [58]; ^8^ Colston et al. [59]; ^9^ Tung et al. [64]; ^10^ Chen et al. [1]; ^11^ Fernández-Bravo and Figueras [2]; ^12^ Bartie et al. [16]; ^13^ Erickson et al. [17].

The placement and nomenclature of *A. dhakensis* as a separate and distinct species has not always been settled. The initial description of this species presented *A. dhakensis* as a subspecies of *A. hydrophila*, termed *A. hydrophila* subsp. *dhakensis,* having been isolated for the first time by Kühn et al. [55] from cases of diarrhoea in children under 5 years old in Bangladesh during 1993–1994 [56]. Later, the application of more discriminatory molecular tools showed that *A. hydrophila* subsp. *dhakensis* grouped together with *Aeromonas aquariorum* (the type strain was MDC47^T^, also named DSM 18362^T^ or CECT 7289^T^), with both sharing a negative arabinose-fermenting phenotype [57]. This discovery culminated in the formal reclassification of *A. hydrophila* subsp. *dhakensis* and *A. aquariorum* as a single species, namely *A. dhakensis* [58]. The type strain of *A. dhakensis* is CIP 107500^T^, i.e., the P21 strain isolated by Kühn et al. [55], which is also referred to in culture collections as DSM 17689^T^, CECT 5744^T^, CCUG 45377^T^, CCM 7146^T^ or LMG 19562^T^ [51,58]. Given the changes in nomenclature, previous studies of *A. aquariorum* and *A. hydrophila* subsp. *dhakensis* can be interpreted as synonymous with the presence of *A. dhakensis*. The increasing availability of whole-genome sequences has further confirmed the delineation of *A. dhakensis* from *A. hydrophila* [16,59,60]. Of course, the correct and consistent use of nomenclature is vital to avoid confusion and misdiagnosis and to allow for the accurate monitoring of epidemiological trends [61]. Figure 1 provides a timeline of milestones in *A. dhakensis* research, particularly relating to nomenclature and classification, as well as increasing recognition for its role in fish and human diseases.

### 2.2. Isolation and Culture Conditions

Selective media are often used for the primary isolation of aeromonads, including *A. dhakensis*, from complex samples such as food, faeces or water, with Rimler-Shotts (RS) agar [65], Aeromonas agar (Ryan formulation; [66]), blood agar supplemented with ampicillin to increase selectivity for aeromonads [67] and glutamate starch penicillin (GSP) agar [48] offering four commonly used medium formulations. Thereafter, non-selective media such as trypticase soy [68] and blood agars [69] are frequently employed for routine subculture or where samples are anticipated to be low in microbial diversity, such as internal organs. Like many mesophilic aeromonads *A. dhakensis* is capable of optimal growth in 0–3% (*w*/*v*) sodium chloride [51], with isolates from aquatic hosts typically cultured between 28 and30 °C [39,48] and human clinical isolates more commonly cultured at 35–37 °C [27,32,69,70], which reflects the standard procedures in different laboratories.

Colonies of *A. dhakensis* form large (ca. 3 mm) convex colonies on agar following 24–48 h incubation at 28 °C, with the precise appearance dependent on the culture medium [5,71,72]. *A. dhakensis* colonies are indistinguishable by eye from many other aeromonads, including *A. hydrophila*, perhaps helping to explain frequent misidentification of *A. dhakensis* as *A. hydrophila.* Certainly, mixed cultures of different *Aeromonas* species from samples have been reported, including fish internal organs [16], which underlines the importance of selecting more than one colony for identification tests, even if colonies appear to be indistinguishable.

### 2.3. Phenotypic Characteristics and Species Identification

Isolates from primary culture on agar can be identified provisionally as *Aeromonas* spp. by colony appearance and the results of Gram staining and several biochemical tests. *A. dhakensis* is a motile Gram-negative rod with cell dimensions of ca. 0.3–1.0 × 1.0–3.5 µm [69]. Like other members of the *Aeromonas* genus, *A. dhakensis* is a facultative anaerobe and tests positive for catalase and oxidase, whilst the species is differentiated from *Vibrio* spp. by exhibiting resistance to the vibriostatic agent pteridine O/129 (150 µg disk) and an inability to grow in 6.5% sodium chloride [73]. Strains of *A. dhakensis* report a 7047125 biochemical profile via API-20E testing [51], and the typical reactions to suites of biochemical tests applied to distinguish *Aeromonas* species have been reported elsewhere [14,48,51,68,74]. Nevertheless, phenotypic markers used in primary identification lack sufficient discriminative power to permit reliable species attribution. Furthermore, the phenotypic attributes of *A. dhakensis* and *A. hydrophila* are remarkably similar, with the L-arabinose negative fermentation reaction and the production of acid from urocanic acid being differential features used to indicate the presence of the former [48,51,56,58,74]; however, these tests are often not applied routinely. Other differential observations in phenotypic studies between *A. dhakensis* and *A. hydrophila* have been noted, with alkyl sulfatase activity more common to *A. dhakensis* [51,75], whilst the production of acid from L-fucose is more typical of *A. hydrophila* [51,56]. Nevertheless, these enzymatic and assimilation tests have been limited to a small number of strains and so traits may not differ reliably across the species. Moreover, atypical phenotypic reactions can occur, as demonstrated by *myo*-inositol-utilising strain variants of *A. dhakensis* isolated from striped catfish (*Pangasianodon hypophthalmus*) in Malaysia [8] and from pabda (*Ompok pabda*) in Bangladesh [12], a characteristic thought to be unique to sequence type (ST)251 hypervirulent isolates of *A. hydrophila* (i.e., vAh) [76].

To compound identification issues, information in databases is often configured for variation in non-representative strains (i.e., human disease-associated isolates) that results in low similarity scores and prevents the correct placement of isolates derived from other sources, such as fish and the environment [77,78]. In addition, there is a risk that not all commercial phenotyping systems are updated sufficiently often to accommodate the recent changes in taxonomy [79]. As a result, the consensus is that phenotypic traits should not be relied upon solely for the definitive species identification of aeromonads [71]. Newer technologies, such as rapid protein fingerprinting via matrix-assisted laser desorption ionization mass spectrometry–time of flight mass spectrometry (MALDI-TOF MS) that are popular and routine in high-throughput diagnostic laboratories may possess improved accuracy when identifying *Aeromonas* species [78], but *A. dhakensis* is still prone to misidentification when using this technique [17,79,80].

Together, these observations mean that the misidentification of species within the *Aeromonas* genus is common, and up to two-thirds of species names provided in the literature obtained via phenotypic testing are estimated to be incorrect [81]. This includes identification errors in important reference resources used routinely to align and speciate unassigned strains, such as PubMLST [https://pubmlst.org/organisms/aeromonas-spp; accessed on 17 March 2024] and the National Center for Biotechnology Information (NCBI) [https://www.ncbi.nlm.nih.gov/; accessed on 17 March 2024] [59,60]. However, the NCBI genome assembly database now has a taxonomy tool to detect erroneous species assignments and flag submissions that fail to meet the 96% average nucleotide identity (ANI) threshold that defines a species [82,83]. This taxonomy checking tool shows that ten isolates submitted as ‘*A. hydrophila*’ and their genome assemblies match more closely the type strain of *A. dhakensis* by ANI rather than *A. hydrophila* (Table 2), which results in suppression of the original RefSeq (i.e., GCF) records. Similarly, the submission of genome data to NCBI has led to three isolates originally described as ‘*A. hydrophila’* to be recently re-assigned to *A. dhakensis* based on ANI values, including one environmental and two clinical isolates (Table 2). Misidentification can cascade to multiple species assignments, with the ST656 isolate from grass carp (*Ctenopharyngodon idella*) sequenced and described as ‘*A. hydrophila*’ GD18 [72] rather than the more likely ‘*A. dhakensis*’ due to comparative genomic analyses by the authors aligning to a previously misidentified isolate ‘*A. hydrophila*’ (L14f; GCF_000813465.1) sampled from lake water in Malaysia [84]; however, more recent ANI analyses and NCBI taxonomic checks place representatives of ST656 within the *A. dhakensis* species [16]. These conflicting descriptions partly explain why *A. dhakensis* may have been overlooked as a major cause of MAS outbreaks and how misidentification can persist. Of course, improvements in the accuracy of the publicly available genome records and appropriate validation will assist with monitoring and assessing the pathogenic threats posed by both *A. dhakensis* and *A. hydrophila* [85,86].

### 2.4. PCR Approaches to Identification

Given the challenges with phenotypic tests for accurate species determination, PCR assays have proved popular for detecting the presumptive presence of DNA motifs from *Aeromonas* spp., especially in research and small-scale diagnostic laboratories. Several PCR assays purportedly targeting sequences specific to *A. hydrophila* have been designed, including primers for genes encoding the aerolysin toxin (e.g., *aerA*; [93,94,95] and 16S rRNA [96], and these are useful in certain circumstances. However, specificity concerns have been raised, particularly the ability of these assays to differentiate *A. hydrophila* and *A. dhakensis*, which may have contributed to the possible under reporting of the latter [17]. Problems with specificity could be inherent from the assay design stage due to nomenclature changes and decisions over the selection of strains included for validation, which may be compounded by errors present in online genomic resources. For example, Trakhna et al. [96] did not include a strain of *A. dhakensis* (or, as then, *A. aquariorum*) in their *16S rRNA* primer validation experiment, which is understandable given the unstable taxonomy at that time, and this exclusion likely explains the inability of the assay to reliably distinguish this species from *A. hydrophila* [16]. Indeed, the *aerA* and *16S rRNA* PCR assays were unable to differentiate *A. hydrophila* and *A. dhakensis* isolates from MAS outbreaks in striped catfish in Vietnam [16]. In addition, Erickson et al. [17] reported that *aerA* PCR was unable to detect all presumptive isolates of *A. hydrophila* (which likely included some *A. dhakensis* isolates), whilst non-specific amplicons can sometimes be generated in samples from other *Aeromonas* spp. (i.e., those that are not *A. dhakensis* and *A. hydrophila*) [16]. To address this lack of simple and reliable identification tools for distinguishing *A. dhakensis* and *A. hydrophila*, Bartie et al. [16] used the core genomes to design primers against the gene encoding a metallo-β-hydrolase (*yjcS*) present in the *A. dhakensis* genome but absent from all available *A. hydrophila* genomes. Initial tests suggested that these primers hold promise to distinguish *A. dhakensis* from *A. hydrophila* and other *Aeromonas* spp. [16], although thorough validation is necessary to comprehensively confirm their usefulness for diagnostic purposes.

**Figure 2:**
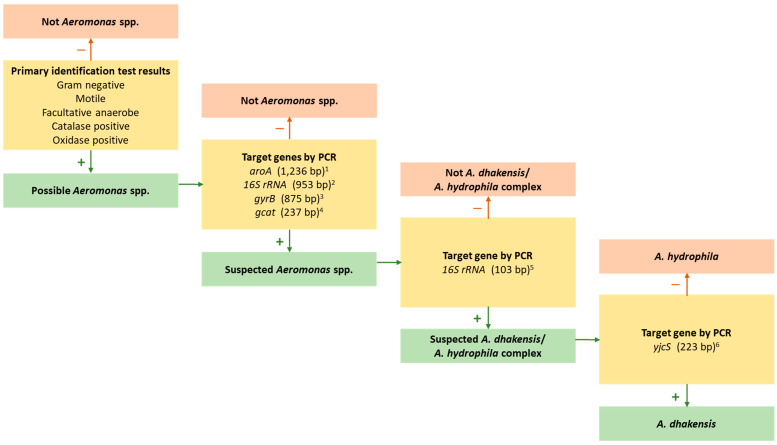
An overview of diagnostic endpoint PCR tests applied to bacteria with primary identification test results consistent with *Aeromonas* spp., and a proposed workflow to assist the differentiation of *Aeromonas dhakensis* from *Aeromonas hydrophila*. ^1^ Cascón Soriano et al. [97]; ^2^ Lee et al. [98]; ^3^ Zhang et al. [99]; ^4^ Latif-Eugenín et al. [100]; ^5^ Trakhna et al. [96]; ^6^ Bartie et al. [16].

A summary of diagnostic PCR tests applied to identify presumptive *Aeromonas* isolates (i.e., exhibiting the expected colony morphology and results from several biochemical tests; Section 2.2 and Section 2.3), and the anticipated test outcomes for *A. dhakensis* and *A. hydrophila*, is presented in Figure 2. Still, caution is advised when applying any PCR assay, and it is best practice to validate primers against representatives of both species before wider use and taking steps to optimise annealing temperatures to confirm specificity and non-amplification of DNA from related species. Further studies with validated *Aeromonas* strains would help to fill gaps within the proposed approach (Figure 2), whilst new PCR assays that differentiate *A. hydrophila sensu stricto* or strains of special importance, such as *A. dhakensis* ST656 that affects striped catfish in Vietnam [16,17], would benefit diagnosis and surveillance efforts. Such additional PCR tools would complement existing primer sets capable of identifying presumptive *Aeromonas* spp. isolates [97,98,99,100], isolates that are either *A. hydrophila* or *A. dhakensis* [96], isolates suspected to be *A. dhakensis* [16], and *A. hydrophila* ST251 [16,76,101], which has caused significant disruption to channel catfish (*Ictalurus punctatus*) culture in the United States, carp production in China, and striped catfish farms in Vietnam [12,16,17,102].

## 3. Phylogeny by Genetic Sequencing

### 3.1. Single-Gene Sequencing

Sequencing of the *16S rRNA* gene fails to discriminate sufficiently within the *Aeromonas* genus to allow for reliable species attribution [103,104], with, for example, only a single base difference in the partial length (ca. 1.4 kb) gene sequence between *A. hydrophila* BACC2 and *A. dhakensis* LMG 19558 [105], and the high similarity of these gene sequences in *A. dhakensis* and *A. caviae* [106]. Instead, sequences of other more variable housekeeping genes have been exploited as single-gene markers to speciate *Aeromonas* spp. isolates, including *A. dhakensis*, such as *gyrB*, *rpoD* [57], *cpn60* (*GroEL*) [107] and *rpoB* [103]. Single-gene sequencing of individual housekeeping genes for aeromonad identification has proven popular for species confirmation due to its relatively low cost, and the utility of these assays and their influence on systematics have been reviewed [108]. Nevertheless, obviously, single genes are not as effective for resolving species as multi-gene approaches [87] and, like other assays, these methods rely on correct species assignments in databases, which is why validated genomes are the preferred source of reference material.

### 3.2. Multi-Locus Approaches

A multi-locus sequence typing (MLST) scheme for *Aeromonas* was devised by Martino et al. [62] based on a comparative sequence analysis of six housekeeping genes, namely *gyrB*, *groL*, *gltA*, *metG*, *ppsA* and *recA*, and this approach can help to inform the overall population structure as well as provide compelling insight into species identification. Notably, analysis of concatenated sequences of five housekeeping genes (*gyrB*, *recA*, *rpoD*, *dnaJ* and *gyrA*) was used to support the reclassification of *A. aquariorum* and *A*. *hydrophila* subsp. *dhakensis* as a single species [58], demonstrating the power of multi-gene analyses. Still, like other databases, the PubMLST *Aeromonas* catalogue [http://pubmlst.org/aeromonas; accessed on 17 March 2024] and database [109] can contain conflicting species descriptors. A pertinent example is PubMLST IDs 742 and 743, recovered from MAS cases in Thailand during 2019, whose genomes, named BT09 and BT06, respectively, were characterised to be ST656 but described on submission to the database as ‘*A. hydrophila*’ and ‘*A. veronii*’, respectively. However, the ribosomal MLST (rMLST) species identification taxonomy check conducted by PubMLST, which extracts the rMLST alleles from genome sequences and compares them to a validated genomic reference database using the ribosomal MLST tool (rMLST; [110]), indicated both genomes to affiliate more closely with *A. dhakensis*, which concurs with the *A. dhakensis* ST656 strain prevalent in Vietnam [16,17].

**Figure 3:**
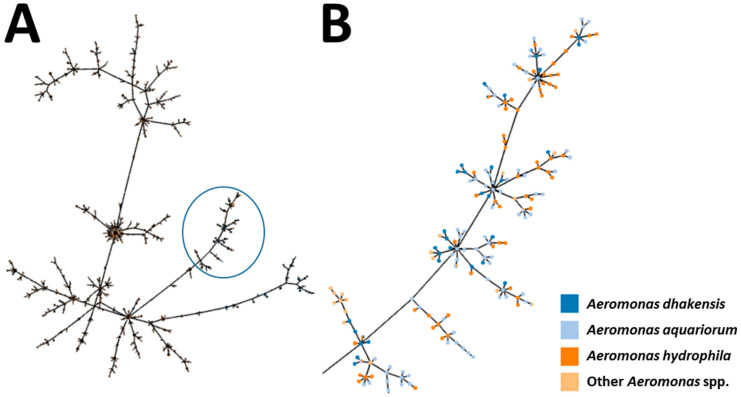
Phylogenetic tree of 2833 *Aeromonas* isolates based on MLST allele sequence similarity using the online PHYLOViZ tool [111]. (**A**): Isolates are colour-coded according to the bacterial species description submitted to the PubMLST *Aeromonas* database (accessed on 19 January 2024), with the branch affiliated to *A. dhakensis* shown with a blue circle; (**B**): enlargement of the circled subset of 293 isolates contained within the *A. dhakensis* branch.

Several MLST studies have examined the phylogenetic relationships of *A. dhakensis*. Novel STs and high genetic diversity were detected amongst 47 clinical isolates of *A. dhakensis* originating from Malaysia, whilst several clonal complexes were detected in a global analysis of 109 isolates, which included the 47 isolates from Malaysia and several isolates from fish hosts such as red drum (*Sciaenops ocellatus*; PubMLST ID 365) and Japanese eel (*Anguilla japonica*; PubMLST ID 371) [87]. Figure 3 presents a phylogenetic tree of 2833 *Aeromonas* isolates found in the PubMLST *Aeromonas* database and a subset of 293 isolates that affiliate most closely to *A. dhakensis* based on allelic profile sequence similarity. The associated epidemiological data, including species identification provided at submission to the PubMLST *Aeromonas* database for the 293 isolates, are found in Table S1, with associated profile data in Table S2. The majority of the isolates included in this phylogenetic branch were recorded as *A. dhakensis* (149 isolates) or the recognised synonym, *A. aquariorum* (89 isolates) (Figure 3). However, 36 isolates were described by submitters as ‘*A. hydrophila*’ or other *Aeromonas* spp. (19 isolates), and many of these isolates share close sequence similarity to the *A. dhakensis* isolates (Figure 3). In eight instances, more than one species is described for the same ST, which can be explained by the change in nomenclature from *A. aquariorum* to *A. dhakensis* [58] for three STs (ST549, ST534 and ST538), with the remaining five STs having conflicting species descriptors that suggests possible species misidentification: ST452 (*A. dhakensis* and *A. caviae*), ST111 (*A. aquariorum* and *A. hydrophila*), ST340 (*A. aquariorum* and *A. hydrophila*), ST590 (*A. dhakensis* and *A. hydrophila*) and ST656 (*A. dhakensis*, *A. hydrophila* and *A. veronii*). More thorough and discriminative genetic analyses, such as whole-genome sequencing (WGS), are required to resolve such conflicts.

### 3.3. Whole Genome Sequencing

The application of WGS and in silico ANI comparisons remains the recommended approach to definitive species identification in the *Aeromonas* genus, and this approach supports the delineation of *A. dhakensis* as a separate species from *A. hydrophila* [16,59,60,79], as do alternative pan-genome phylogenetic methods [112]. At present, relatively few annotated genomes of *A. dhakensis* are available publicly in NCBI (n = 148) compared to *A. hydrophila* (n = 344) (accessed on 5 January 2024). Of these 148 sequences submitted (accessed on 22 January 2024), most derive from isolates collected from human clinical cases or fish hosts in Asia, with fewer data for isolates sampled from the environment (Table S3). The 59 genomes from fish-derived isolates of *A. dhakensis* are predominated by those collected from hosts of high economic significance to aquaculture, which consist of 43 isolates from striped catfish in Vietnam (Table 3), an atypical isolate (*A. dhakensis* 1P11S3) from striped catfish Malaysia [113], seven isolates from Nile tilapia (*Oreochromis niloticus*) in Brazil and Mexico [114], and single isolates from channel catfish (*Ictalurus punctatus*) in the United States, Asian seabass (*Lates calcarifer*) in Canada, and *Ancherythroculter nigrocauda* (a cyprinid) and grass carp (*Ctenopharyngodon idella*) from China. Other *A. dhakensis* isolates with genomes that have derived from fish hosts include an isolate from the skin of an Indian oil sardine (*Sardinella longiceps*), the former *A. aquariorum* type strain (i.e., CECT 7289) cultured from the aquarium water of ornamental fish in Portugal [48], two *A. dhakensis* isolates from redtail catfish (*Phractocephalus hemioliopterus*) in Brazil, and a final commensal isolate (*A. dhakensis* b2-10) from the gut of experimental zebrafish [115] (Table 3). The genome of *A. dhakensis* TN14 [16] has been designated by NCBI to be the reference genome of the species and it was the fiftieth *A. dhakensis* genome submitted to this database (Figure 2).

## 4. Pathogenicity and Virulence of *A. dhakensis*

### 4.1. Comparative Virulence of A. dhakensis and A. hydrophila

Although most virulence studies have centred on *A. hydrophila* as the suspected primary aetiological agent of MAS, mounting evidence suggests that *A. dhakensis* may be equivalent to or even more virulent than *A. hydrophila* counterparts in healthcare settings [2,77]. Indeed, compared to *A. hydrophila* and other aeromonad species, it is intriguing that *A. dhakensis* has been proposed to be more virulent based on virulence gene profiles, a greater prevalence in human *Aeromonas* spp. infections, and greater mortality in mono-microbial cases of bacteraemia [1,2,77,116]. In aquatic hosts, the greater virulence of *A. dhakensis* is supported by anecdotal evidence of *A. dhakensis* isolates being recovered more prevalently than *A. hydrophila* from striped catfish showing signs of MAS in the Mekong Delta [16,17]. In further support, experimental challenge data presented in the patent application for the ALPHA JECT^®^ Panga 2 commercial vaccine for MAS [64] showed striped catfish injected with four representatives of ‘*A. hydrophila* Biotype A’, including AL 20133, which is identical by *gltA* and *metG* sequences to the *A. dhakensis* ST656 strains characterised by Bartie et al. [16] and Erickson et al. [17], caused greater mortality compared with groups infected with two strains of ‘*A. hydrophila* Biotype B’, including AL 20215, which has identical *gltA* and *metG* housekeeping gene sequences to *A. hydrophila* ST251 (Figure S1; Table S4).

### 4.2. Mechanisms of Virulence

Relatively little attention has been paid to determining the mechanisms of virulence of *A. dhakensis* specifically but, given the intimate genetic relatedness of this species to *A. hydrophila*, it can be assumed that many key mechanisms are conserved across these two species and the genus more generally. The main virulence factors studied in aeromonads have been those associated with host colonisation and the processes of adhesion and biofilm formation, as well as toxins and degradative enzymes and the quorum-sensing and secretory systems that control their expression and delivery [1,2,71,86,117,118]. The aerolysin toxin is probably the most well-known and best-characterised cytotoxin of *A. dhakensis* and *A. hydrophila*, and this virulence factor, in addition to the ahh1 haemolysin, assists in overwhelming the host immune defences [1,119,120,121]. The virulence capabilities of *A. dhakensis* isolates from fish hosts has been largely limited to screening for the presence or absence of predicted virulence genes [86,87,122,123], although challenge models have been established for several aquatic organisms (Table 4). Fortuitously, studies conducted with ‘*A. hydrophila*’ GD18 from diseased grass carp have provided a valuable insight into the virulence of *A. dhakensis* (the species to which this isolate is suspected to belong), and findings have highlighted the role of VasH, a transcriptional regulator of the type VI secretion system (T6SS), in cytotoxicity assays and challenge trials [72]. Furthermore, Li et al. [124] provided evidence for the importance of another transcription factor in the pathogenicity of the GD18 strain, specifically ferric uptake regulator (Fur), which plays a role in activating the T6SS. Many of the virulence factors important for *A. dhakensis* to infect aquatic hosts may play roles in human disease too, and for thorough insights into virulence mechanisms, readers are guided to Chen et al. [1], in addition to recent articles on the importance of flagellar-mediated motility [125], the UvrY [126] and KdpE transcriptional factors [127], and the VgrG mediator of T6SS toxin effectors [128].

### 4.3. Strains with Heightened Virulence

An opportunistic pathogen is predicted to exhibit a sporadic epizootic pattern of disease, and indeed, many *Aeromonas* spp. are limited to defined local outbreaks with varied strain types responsible for disease [130,131]. However, exceptions within the *Aeromonas* genus have been noted, with the hypervirulent *A. hydrophila* ST251 providing a pertinent example, causing MAS infections in fish hosts worldwide, including in the United States, China and Vietnam [12,16,17,102]. Similarly, clonal restriction was detected in the *A. dhakensis* isolates recovered from striped catfish in Vietnam, with ST656 isolates predominantly associated with the mortality cases investigated and the data suggesting clonal expansion of this strain within the Mekong Delta region since at least 2013 [16]. ST656 isolates, suspected to be *A. dhakensis*, have also been cultured from fish hosts in Thailand (Section 3.2), which suggests that this clone could become a problem in a similar way to *A. hydrophila* ST251, although this requires further investigation. Taken together, this evidence raises the prospect that certain clonal types of *Aeromonas* spp. possess selective advantages that allow them to expand and affect intensive aquaculture operations in a manner distinct from other strains that are only able to trigger sporadic outbreaks, which challenges the dogma that motile *Aeromonas* spp. are typically opportunists. Enhanced detection of *A. dhakensis*, including as a zoonotic agent, should encourage more genomes to be submitted, and this will assist in the determination of problematic strains such as ST656. This enriched genomic information will facilitate a closer examination of strain-specific regions that may explain their advantage and success [12,16,132].

## 5. Prevention and Treatment of MAS Cases Caused by *A. dhakensis*

Like many other infectious diseases in fish, the prevention of MAS relies on appropriate health management of stocks, which includes good husbandry, the avoidance of stress such as that caused by overcrowding and handling, and adherence to the key principles of biosecurity [13,117,123]. In certain circumstances, vaccination against *Aeromonas* spp., including *A. dhakensis*, can be a suitable approach to protect against these pathogens [133]. In Vietnam, the multivalent injectable commercial vaccine (ALPHA JECT^®^ Panga 2; Pharmaq) has been available since 2016 for striped catfish to protect against MAS and enteric septicaemia caused by the bacterium *Edwardsiella ictaluri*. The antigens in this inactivated whole-cell vaccine formulation are *E. ictaluri* AL 20 658 and two biotypes of ‘*A. hydrophila*’ but, as described above, Biotype A is possibly an *A. dhakensis* ST656 isolate, whilst Biotype B may be *A. hydrophila* ST251. Thus, the current vaccine formulation should offer appropriate coverage against the two most prevalent circulating MAS-causing strain types affecting catfish farms in the Mekong Delta region since 2013 [16,17]. Still, continual strain surveillance is required to ensure that vaccines continue to provide appropriate protection, and the contribution of other motile *Aeromonas* spp. to MAS outbreaks warrants further attention [16].

## 6. Antibiotic Resistance

When MAS outbreaks occur, antibiotic therapy by metaphylaxis is often the only treatment option, with the agents administered commonly in the feed [134,135]. However, every application of antibiotics risks the possible emergence and selection of resistance and fish farms experiencing MAS outbreaks are no exception [136,137]. Due to the prodigious ability of aeromonads, including *A. dhakensis*, to disseminate genes by horizontal gene transfer, there is a risk that antibiotic-resistance genes selected in strains in aquaculture environments will move into strains and pathogens capable of causing serious infections in humans [138]. Indeed, environmental isolates, as well as some derived from fish, have been implicated as a reservoir for the multidrug resistance elements [139], and, given its almost ubiquitous presence in freshwater environments, *Aeromonas* has been proposed as an indicator genus to study antimicrobial resistance from a One Health perspective [140,141].

Species-specific antibiotic resistance profiles have been noted in *Aeromonas* spp. collected in aquaculture settings [142], with strains of *A. dhakensis* being no exception. *A. dhakensis*, like *A. hydrophila*, carries intrinsic resistance genes to beta-lactams (*blaAQU* and *blaOXA*) and to later generations of cephalosporins, notably *cphA* for reduced susceptibility to carbapenem [80,116]. In addition, strains of *A. dhakensis* containing *blaCTX-M* that confer reduced susceptibility to extended-spectrum β-lactamases (ESBLs) have been reported in aquaculture-derived isolates in Korea [143], whilst isolates associated with MAS outbreaks in Vietnam contained genes encoding reduced susceptibility to sulphonamide (*sul1*), tetracycline (*tetA*) and trimethoprim (*dfrA1*) [16]. Of particular concern is the detection of the *mcr-1* colistin resistance gene in *A. dhakensis* [33], which confers resistance against this drug of last resort [144]. Like other aeromonads, *A. dhakensis* may carry resistance genes on mobile plasmids [145] or be integrated into bacterial genomes by class 1 integrons [146].

High multiple antibiotic resistance (MAR) indices (i.e., >0.2) have been reported for *A. dhakensis* isolates from various aquaculture settings in Africa and Asia, including ornamental fish species [7,52,147] and from farms culturing tilapia [145], African catfish (*Clarias gariepinus*) [41], and striped catfish [8], which implies the application of various antimicrobials in these settings [148]. However, it should be noted the adoption of standardised antimicrobial testing methods is not universal in studies of isolates from aquatic hosts, and the definition of reduced susceptibility or non-wild type strains varies and may be based on epidemiological cut-off values not validated for *A. dhakensis* [149]. Notably, standardised antibiotic susceptibility methods for testing isolates derived from aquatic hosts have been proposed for *A. hydrophila* [149], but similar studies are warranted for *A. dhakensis*.

In aquaculture, similar to human medicine [3,150,151], the presence of genetic determinants in *Aeromonas* spp. isolates that confer reduced susceptibility or resistance to antimicrobial agents is a concern, as it can lead to delays in administering effective treatments [152]. In human systemic infection, differential diagnosis of *A. dhakensis* from *A. hydrophila* is valuable [116] given the heightened risk of antimicrobial resistance involving carbapenem resistance [153,154]. Equally, increased minimum inhibitory concentration (MIC) values in *A. dhakensis* relative to *A. hydrophila* have been reported for isolates derived from fish [17] and human patients [77], which stresses the need for accurate diagnoses.

## 7. Conclusions

This review provided a synthesis of the key literature concerning the role of *A. dhakensis* as a pathogen of aquatic species, especially in aquaculture settings, with a primary focus on approaches to identify this bacterium accurately and permit its differentiation from the closely related species, *A. hydrophila*. Changes in species nomenclature, in addition to inherent limitations associated with some common tests and resources employed for identification, may have led to the underappreciation of the problems caused by *A. dhakensis* in aquaculture as well as in the clinic. Improvements in the effectiveness of identification methods, such as those proposed in Figure 2 and the accuracy of various commercial databases through the inclusion of more validated strains will allow for greater recognition of this opportunistic pathogen and encourage the development and implementation of effective approaches to infection prevention and control. The wide geographic and host range of *A. dhakensis* (Table 1), in addition to the occurrence of strains with reduced susceptibility and resistance to several classes of antimicrobial agent, renders this bacterium a threat to the concept of One Health. Whilst reports of the same strains infecting humans and aquatic animals remain rare [87], improved identification of *A. dhakensis* and an expansion of genetic information will permit improved appreciation for the zoonotic risk posed by this species [155]. Although it is too early to conclude that *A. dhakensis* is more virulent in aquatic animals than *A. hydrophila* counterparts, the evidence for this proposal is mounting, and it would be consistent with present understanding in human clinical cases, with systematic surveys and challenge studies required to strengthen this suggestion. In aquaculture, further studies to characterise *A. dhakensis* isolates from MAS cases will allow a greater understanding of the molecular epidemiology of the most important strains, with recent studies indicating the possible presence of a strain with increased virulence. Still, further research is required to determine whether *A. dhakensis* ST656 poses a threat to aquaculture operations in a similar way to that posed by *A. hydrophila* ST251. Finally, this review suggests an approach to assist the accurate identification *A. dhakensis* (Figure 2), and it provides an impetus to screen existing strain collections to reveal the true prevalence and impact of this zoonotic species in aquatic hosts, the environment and in healthcare settings.

## Figures and Tables

**Table 1:** Reported isolations of *Aeromonas dhakensis* from apparently diseased farmed aquatic hosts and ornamental and wild fish hosts, including the country of origin and year of isolation. To date, *A. dhakensis* has only been isolated from warm-water aquatic species.

Host or Sample Type	Country of Isolation	Year of Isolation ^a^	Reference
**Farmed hosts**			
*Anabas testudineus* (Climbing perch)	Vietnam	2019	[37]
*Anguilla japonica* (Japanese eel) ^b^	South Korea	[2013]	[38]
*Clarias batrachus* (Walking catfish) ^b^	Philippines	2013	[39]
*C. batrachus*	Malaysia	2019	[8]
*C. batrachus*	Indonesia	[2021]	[40]
*Clarias gariepinus* (African catfish)	Nigeria	2019–2020	[41]
*Colossoma macropomum* (Tambaqui)	Brazil	[2020]	[4]
*Labeo catla* (Catla)	India	2020	[42]
*Macrobrachium rosenbergii* (Giant freshwater prawn)	China	2021	[24]
*Oncorhynchus mykiss* (Rainbow trout)	Turkey	2013	[43]
*O. mykiss*	Peru	[2021]	[44]
*Oreochromis niloticus* (Nile tilapia)	Mexico	2009	[45]
*O. niloticus*	India	2018–2019	[46]
*O. niloticus*	India	[2020]	[46]
*Oreochromis* spp. (Tilapia)	Malaysia	2019	[8]
*Piaractus mesopotamicus* (Pacu)	Brazil	[2016]	[47]
*Pangasianodon hypopthalmus* (Striped catfish)	Vietnam	2013–2019	[16]
*P. hypopthalmus*	Vietnam	2017–2021	[17]
*P. hypophthalmus*	Malaysia	2019	[8]
**Ornamental fish hosts**			
Aquaria of unspecified ornamental fish ^b^	Portugal	2004–2005	[48]
*Carassius auratus* (Goldfish)	Turkey	2014	[43]
*Cyprinus carpio* (Koi)	Sri Lanka	2007–2008	[49]
*Osphronemus goramy* (Giant gourami)	Sri Lanka	2007–2008	[49]
*Otocynclus affinis* (Dwarf suckermouth catfish)	Peru	2021	[50]
*Poecilia reticulata* (Guppy)	Sri Lanka	2020–2021	[7]
*Trachelyopterus galeatus* (Driftwood catfish)	Peru	2021	[50]
**Wild hosts**			
*Anguillicola crassus* (European eel)	Spain	2004–2005	[51]
*Dawkinsia assimilis* (Mascara barb)	India	2018	[52]
*Ompok pabda* (Pabda)	Bangladesh	[2023]	[12]
*Sardinella longiceps* (Indian oil sardine)	India	[2016]	[53]

^a^ Where the year of isolation is unknown, the year of publication is provided in square brackets instead. ^b^ Described initially as ‘*Aeromonas aquariorum*’.

**Table 2:** *Aeromonas dhakensis* genomes originally submitted to the National Center for Biotechnology Information (NCBI) database as ‘*Aeromonas hydrophila’* that may have been misidentified. NCBI taxonomy checking tool status and average nucleotide identity (ANI) similarity to the *A. dhakensis* and *A. hydrophila* type strains are provided. Ten genomes listed fail the taxonomy check, and as such, the GCF records are suppressed; meanwhile, the species name of the other three genomes was corrected based on ANI from ‘*A. hydrophila*’ to ‘*A. dhakensis*’ in 2018.

Submitted Species Name	Isolate Name	Sample Type	Taxonomy Check Status	ANI against *A. hydrophila* ATCC 7966^T^ (%)	ANI against *A. dhakensis* CIP 107500^T^ (%)	Assembly Accession	Reference
**Records with apparent mismatch to original submitted species name**							
*Aeromonas hydrophila*	116	Human	Mismatch	93.28	97.28	GCF_000350405.1	[87]
*A. hydrophila*	187	Human	Mismatch	93.10	97.35	GCF_000354635.1	[87]
*A. hydrophila*	14	Human	Mismatch	93.24	97.16	GCF_000354655.1	[84]
*A. hydrophila*	259	Human	Mismatch	93.13	97.38	GCF_000354695.1	[84]
*A. hydrophila*	145	Human	Mismatch	93.31	97.35	GCF_000586035.1	[87]
*A. hydrophila*	L14f	Lake water	Mismatch	93.27	97.36	GCF_000813465.1	[84]
*A. hydrophila*	YL17	Compost	Mismatch	93.26	97.34	GCF_000612075.2	[88]
*A. hydrophila*	B11	*Anguilla japonica* (Japanese eel)	Mismatch	93.31	97.36	GCA_013205705.1	[89]
*A. hydrophila*	BB1457	Hospital wastewater	Mismatch	93.12	97.33	GCA_903684605.1	[90]
*A. hydrophila*	AYN7	*Heteropneustes fossilis* (Asian stinging catfish)	Mismatch	93.42	97.28	GCA_028771245.1	PRJNA911000
**Records with recently corrected species assignments**							
*Aeromonas dhakensis*	173	Human	Species match	N/A	97.45	GCF_000354675.1	[87]
*A. dhakensis*	SSU	Human	Species match	N/A	97.38	GCF_000298055.1	[91]
*A. dhakensis*	KOR1	*Kandelia obovate* (Mangrove)	Species match	N/A	97.41	GCF_001306015.1	[92]

**Table 3:** Whole genomes of *Aeromonas dhakensis* isolates sampled from fish hosts that have been sequenced and are publicly available.

Host or Sample Type	Isolate Name	Country of Isolation	Year of Isolation	Assembly Accession	Bioproject	Reference
Aquarium water	CECT 7289	Portugal	2003	GCF_000819705.1	PRJEB7020	[48]
*Ancherythroculter nigrocauda* (Cyprinid)	202108B1	China	2021	GCF_034143565.1	PRJNA1009974	
*Ctenopharyngodon idella* (Grass carp)	202108C2	China	2021	GCA_035658375.1	PRJNA1060866	
*Danio rerio* (Zebrafish)	b2-100	China	2019	GCF_023920205.1	PRJNA797204	[115]
*Ictalurus punctatus* (Channel catfish)	OTH-19-VL-NY-MS-0027	United States	2019	GCA_020765715.1	PRJNA481355	
*Lates calcarifer* (Asian seabass)	OTH-21-VL-ON-ON-0001	Canada	2021	GCA_032496525.1	PRJNA503849	
*Oreochromis niloticus* (Nile tilapia)	Aer_On15M	Brazil	2009	GCF_017163915.1	PRJNA607226	
*O. niloticus*	Aer_On24M	Brazil	2009	GCF_017310095.1	PRJNA594314	
*O. niloticus*	26M	Brazil	2009	GCF_019348695.1	PRJNA590952	
*O. niloticus*	CAIM 1873	Mexico	2009	GCF_003989145.1	PRJNA422283	[114]
*O. niloticus*	OnIF3	Brazil	2010	GCF_018094765.1	PRJNA591217	
*O. niloticus*	IF_2	Brazil	2010	GCF_019348645.1	PRJNA590791	
*O. niloticus*	Aer_OnIF1	Brazil	2010	GCF_022703095.1	PRJNA577584	
*Pangasianodon hypophthalmus* (Striped catfish)	33 isolates, e.g., 12-AH41	Vietnam	2012-2022	e.g., GCA_031915985.1	PRJEB65955	
*P. hypophthalmus*	10 isolates, e.g., TN14	Vietnam	2013-2018	e.g., GCF_905132925.1	PRJEB41556	[16]
*P. hypophthalmus*	1P11S3	Malaysia	2019	GCF_015666195.1	PRJNA679132	[113]
*Phractocephalus hemioliopterus* (Redtail catfish)	Aer_Pi12.1HTAS	Brazil	2009	GCF_025266835.1	PRJNA595107	
*P. hemioliopterus*	Pi16.2MC	Brazil	2009	GCF_018094645.1	PRJNA591201	
*Sardinella longiceps* (Indian oil sardine)	F2S2-1	India	2015	GCF_001673685.1	PRJNA312130	[53]

**Table 4:** Experimental challenge models established with whole bacterial cells of *Aeromonas dhakensis* and extracellular products that have been shown to be virulent and cause disease signs in aquatic hosts.

Host	Challenge Material	Delivery	Reference
*Anguilla anguilla* (European eel)	Whole bacterial cells	Intraperitoneal injection (i.p.)	[51]
*Danio rerio* (Zebrafish)	Whole bacterial cells	i.p.	[72] ^a^, [127]
*Labeo rohita* (Rohu)	Whole bacterial cells	i.p.	[52]
*Macrobrachium rosenbergii* (Giant freshwater prawn)	Whole bacterial cells	Intramuscular injection (i.m.)	[24]
*Oncorhynchus mykiss* (Rainbow trout)	Whole bacterial cells; Extracellular products (ECPs)	i.p.; i.m.	[105]
*Oreochromis mossambicus* (Mozambique tilapia)	Whole bacterial cells; ECPs	i.p	[45]
*Oreochromis niloticus* × *O. mossambicus* (Hybrid tilapia)	Whole bacterial cells; ECPs	i.p.	[114]
*O. niloticus* × *O. mossambicus*	Whole bacterial cells	i.p.	[129]
*Pangasianodon hypophthalmus* (Striped catfish)	Whole bacterial cells	i.p.	[64]
*Piaractus brachypomus* (Pacu)	Whole bacterial cells	i.p.	[47,50]
*Solea vulgaris* (Dover sole)	ECPs	i.p.	[45]

^a^ Challenge isolate described as ‘*A. hydrophila*’ GD18 but suspected to be *A. dhakensis*.

## Data Availability

All data are available in the Supplementary Materials or by request to the Corresponding Author.

## Supplementary Materials

The following supporting information can be downloaded at: https://www.mdpi.com/article/10.3390/pathogens13060465/s1, Figure S1: Phylogram of 19 *Aeromonas* spp. isolates based on concatenated *gltA* and *metG* sequences using the Clustal Omega sequence alignment tool [156]; Table S1: Epidemiological data and species identification provided at submission to the PubMLST *Aeromonas* database for 293 isolates that affiliate most closely to *Aeromonas dhakensis* based on allelic profile sequence similarity (accessed on 19 January 2024); Table S2: Multi-locus sequence typing (MLST) allelic profiles of the 188 distinct sequence types in the PubMLST *Aeromonas* database associated with 293 isolates that affiliate most closely to *Aeromonas dhakensis* based on allelic profile sequence similarity (accessed on 19 January 2024); Table S3: Key information associated with the 148 annotated genomes available in the NCBI database for *Aeromonas dhakensis*; Table S4: Summary of *gltA* and *metG* housekeeping gene sequences for ‘*Aeromonas hydrophila*’ Biotype A and Biotype B (isolate AL 20215) found in the ALPHA JECT^®^ Panga 2 commercial vaccine, 13 *Aeromonas* spp. isolates of known sequence type (ST) selected as reference isolates in the vaccine patent application, and *A. hydrophila* ST251 and *A. dhakensis* ST656 isolates from recent MAS outbreaks affecting striped catfish in Vietnam.
