# Supplementary material for: Aeromonas dhakensis: A Zoonotic Bacterium of Increasing Importance in Aquaculture
Source: Pathogens. 2024 May 31;13(6):465. doi: 10.3390/pathogens13060465 (PMC11207067; doi:10.3390/pathogens13060465)
Supplement: Supplementary file 1 [file pathogens-13-00465-s001.zip › Supplementary Figure 1.pdf]

pathogens

MDPI

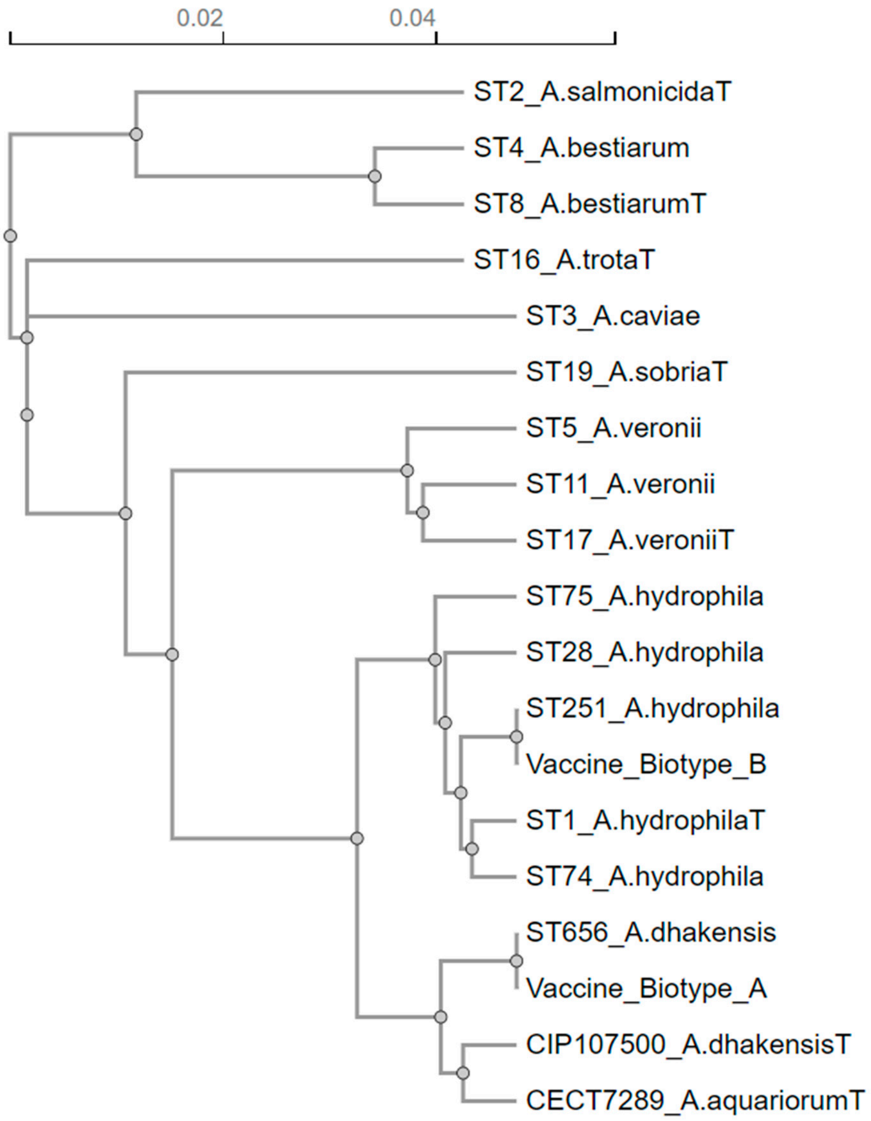

Figure S1. Phylogram of 19 *Aeromonas* spp. isolates based on concatenated *gltA* and *metG* sequences using the Clustal Omega sequence alignment tool [156]. The tree includes '*Aeromonas hydrophila*' Biotype A (isolate AL 20133) and Biotype B (isolate AL 20215) found in the ALPHA JECT® Panga 2 commercial vaccine, 13 *Aeromonas* spp. isolates of known sequence type (ST) selected as reference isolates in the vaccine patent application [64], and *A. hydrophila* ST251 and *A. dhakensis* ST656 isolates from MAS outbreaks affecting striped catfish in Vietnam [16,17]. The concatenated sequence of the '*A. hydrophila*' Biotype A isolate is identical to that of the *A. dhakensis* ST656 sequences, whilst the *A. hydrophila* Biotype B isolate shares identical sequence to *A. hydrophila* ST251 isolates [16,17].
